# When do health and social care practitioners consider digital healthcare to be ‘good care’ for people with co-occurring alcohol-use disorder and depression? a qualitative analysis of practitioners’ accounts

**DOI:** 10.1186/s12913-025-13883-3

**Published:** 2025-12-23

**Authors:** Katherine Jackson, Eileen Kaner, Lucy Yardley, Amy O’Donnell

**Affiliations:** 1 Population Health Sciences Institute, Newcastle University, Newcastle Upon Tyne, UK; 2 School of Psychological Science, University of Bristol, Bristol, UK; 3 School of Psychology, University of Southampton, Southampton, UK

**Keywords:** Alcohol-use disorder, Mental health, Health and social care practitioners, Care, Relationality, Digital heath

## Abstract

**Background:**

People with alcohol-use disorder (AUD) and depression may experience a range of barriers to accessing formal healthcare; utilising digital technologies has been proposed as one way to improve provision for this population. The COVID-19 pandemic rapidly expanded the use of digital health in mental health and substance use services, which had previously been slow to implement telemedicine or m-health approaches. This study aimed to explore when health and social care practitioners, who work with people with AUD and depression, consider digital healthcare, in particular telemedicine and m-health to be ‘good care’. The work has implications for the development and implementation of digital healthcare for this population.

**Methods:**

A qualitative study was carried out using semi-structured interviews with 26 health and social care practitioners in the North-East and North Cumbria area of England, UK, who work with people with AUD and depression. Purposive and snowballing sampling approaches were adopted. Data were analysed inductively using thematic analysis and then framed using concepts from Ethics of Care Theory, principally, Tronto and Fisher’s four phases and ethical elements of ‘good care’.

**Results:**

Themes were grouped into the four phases/ elements of care: Attentiveness (digital exclusion / inclusion, safety); Responsibility (in addition to a wider infrastructure of in-person support); Competence (embodiment and physicality, flexible and adaptable); and Responsiveness (honesty, and motivation). The findings also highlighted the importance of considering the relational contexts of people with AUD and depression.

**Conclusions:**

There are some areas where practitioners perceive that digital healthcare can be valuable for people with AUD and depression. For example, it can be more flexible than current provision, it can be inclusive for some people, and it can offer a level of anonymity. However, for digital healthcare to be considered to provide good care, there should be attentiveness to people’s material and relational circumstances, and their levels of digital literacy, to ensure inequalities in this population are not exacerbated.; it should always be part of a wider package of in-person relational support. Attention should always be given to safety features and planning.

**Supplementary Information:**

The online version contains supplementary material available at 10.1186/s12913-025-13883-3.

## Introduction

People who drink alcohol heavily and / or experience alcohol dependence (from here referred to as having an alcohol-use disorder or AUD) [1] commonly also have depression [2, 3], and are known to be at a much greater risk of suicide than the general population [4]. Individuals who have experienced trauma and/or who live in areas of socio-economic deprivation, are more likely to be affected by both conditions [5–9]. Despite the common co-occurrence of AUD and depression, and the inequalities this population often face, current formal health and social care provision for people who experience these conditions together is often inadequate, with evidence of a longstanding global care gap [10–12]. In England, United Kingdom (UK), care for this population is provided by a varied workforce including community alcohol and drug services, primary and secondary healthcare, mental health services and the community and voluntary sector organisations. Multiple factors compromise appropriate care provision. These include stigma, lack of an adequately trained workforce, limited financial investment in both alcohol and mental health services, and the differing and even conflicting, professional, cultural, and structural paradigms under which the various health and social care providers operate [12–14]. Digital health technologies have been positioned in policy as one potential response to closing the treatment gap [15–19]. Telemedicine (using telecommunication and information technology to connect patients and providers across geographical distances in real time to provide assessments and therapeutic interventions [20]) and m-Health (use of mobile communication devices such as smartphones and tablet computers for healthcare [21]) could, theoretically, address capacity and access issues in the current care system by providing more flexible, tailored, integrated provision, that is scalable at relatively low cost [22–27]. Prior to the COVID-19 pandemic health and social care services for people with AUD and depression were delivered mainly face-to-face either one-to-one or in groups. However, the social distancing measures implemented during the pandemic led to a rapid shift to telemedicine provision, by formal health and mental healthcare services, community drug and alcohol services and mutual aid groups (e.g. Alcoholics Anonymous) in England and elsewhere [28–30].

Empirical research has explored views on and experiences of implementing digital healthcare including telemedicine and m-health within alcohol and/or mental health services. Advantages highlighted in this literature include the potential for digital technologies to support improved information sharing, medication, and condition self-management [31]. However, as well as highlighting a need for improved access to training in digital competencies [32, 33], care practitioners have expressed concerns around: the accuracy of remote psychological assessments due to the challenges of detecting non-verbal cues; lack of control over the assessment context; and tendency to focus on algorithm-driven rather than person-based needs [34, 35]. Linked to this point, existing evidence also highlights providers’ concern that digital technology could also mean the loss of the embodied dimension required for therapeutic relationship building [36], resulting in more transactional as opposed to relational care [35]. A recent rapid evidence review by The Health Foundation found mixed impacts on staff time, depending on the type of technology, due to workflow and task efficiency, usability and skill, and/or the wider implementation context [37]. Some patients have expressed a preference for online as opposed to in-person care delivery, due to the ease of access and relative anonymity this can provide [31, 38]. Yet concerns have also been raised as to the appropriateness of digital healthcare for people with complex needs and high vulnerability such as those with co-occurring AUD and mental ill-health. Challenges include perceived risks around maintaining the security and confidentiality of sensitive personal data collected and stored remotely [31, 38], and evidence that digital exclusion is contributing to disparities in engagement with digital technologies [39, 40].

Indeed, people with mental ill-health and/or substance use conditions are known to experience more digital exclusion than the general population [41, 42]. Digital exclusion is used here to refer to being excluded from accessing or using digital technologies due to factors beyond an individual’s control [43]. Factors impacting digital exclusion include material or financial barriers which mean people lack physical access to technologies and/or the data required to facilitate their use [44]. Digital literacy, which relates to people’s ability to use digital technologies and have the digital skills to improve their lives [45], also shapes access, with people on low incomes, women and older people being most likely to have poor digital skills compared to other groups [44, 46] Other factors affecting digital exclusion in people with mental health and / or substance use conditions include low trust in digital technologies [47], low motivation for self-care [48] and/or low motivation to change [48, 49]. Digital inclusion is the opposite of exclusion, and refers to an individual having sufficient support, skills and literacy to meaningfully access and use the technology required, as well as the appropriate hardware, software, and associated services [50]. When discussing digital inclusion specifically, we therefore refer to the National Digital Inclusion Alliance definition of ‘activities necessary to ensure that all individuals and communities, including the most disadvantaged, have access to and use of Information and Communication Technologies’ [51].

While the existing body of work about barriers to implementation of telemedicine or m-health initiatives provides helpful insights around which factors appear to affect current take-up or delivery, most studies overlook the moral dimensions of how practitioners might perceive digital healthcare for people with co-occurring AUD and depression. Namely, where they might perceive digital healthcare to provide good care, and their concerns about its adequacy or appropriateness for the population they work with. Other studies have found that practitioners’ perceptions and personal experiences that digital healthcare can be beneficial for patients increases their motivation to use or engage with these technologies [52]. Thus, our overall aim is to draw on empirical data from health and social care practitioners who work with people with co-occurring AUD and depression to explore their views about what constitutes good digital healthcare for this population, and to make recommendations about implications for the future development and implementation of digital healthcare in this area of practice. We recognise that what constitutes a framing of ‘good’ care, including digital healthcare, is ill-defined and can be viewed from multiple perspectives [53, 54]. Therefore, we drew on ideas from ethics of care theory during the analysis stage for this study and used Tronto and Fishers conceptualisation of the four ethical and moral dimensions of ‘good care’ [55, 56] to structure the findings. The theory is introduced below, and the methods section provides more detail of how it was applied during the analysis process.

## Theoretical framework

**Fig. 1:**
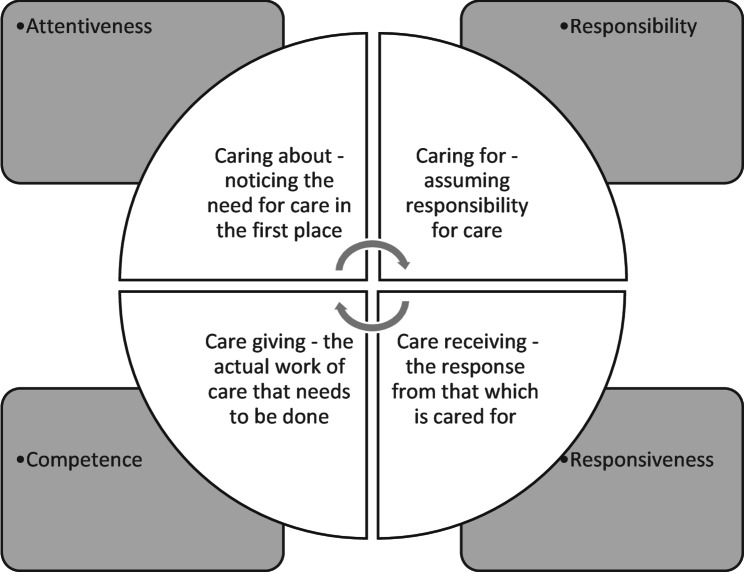
Tronto and Fisher’s original four phases of care and their ethical dimensions [55, 56]

Ethics of Care Theory is a broad area of scholarship [57], and several key tenets are helpful for our argument. Firstly, ethics of care is underpinned by a recognition that people are relational and interdependent; secondly, it recognises that what constitutes ‘good care’ practices are shaped by social contexts; and thirdly, ethics of care scholarship is concerned with social justice and illuminating power imbalances in care practices [57, 58]. Almost, forty years ago, working from a feminist and political perspective and challenging the notion that care is purely an emotion innate to women, Tronto and Fisher [55], proposed a multi-dimensional framing of care, to illustrate it’s complexity. They articulated that within an ethic of care, for care to take place, four phases are required: (1) ‘caring about’ noticing a need for care; (2) ‘caring for’ or taking responsibility for care; (3) ‘care giving’ which is the actual work of care that we think of when we most think of care; and (4) ‘care receiving’ where the person who is being cared for evaluates whether care has taken place [55, 56]. Tronto later articulated that alongside these phases are four moral principles likely to come close to the idea of ‘good’ care: attentiveness, responsibility, competence and responsiveness [56]. Tronto notes that these phases and ethical dimensions, illustrated in Fig. 1, are meant to be helpful for making an assessment about what might constitute good care but are not meant to be prescriptive [59]. More recently, Tronto has expanded the care process to include a fifth phase ‘caring with’ and associated moral dimension ‘solidarity’ which highlights the need to work together to achieve ‘good care’ with a commitment to justice and equality [59].

Some scholars, have specifically applied ethics of care theory to the area of digital healthcare technologies [60]. Here, Pols and colleagues have illustrated the complex ways that patients, their families/ friends and practitioners engage with digital health technologies in different healthcare settings [61, 62]. A key insight is the varied ways people engage with digital health technologies (i.e. for some it can be very beneficial while others struggle to engage), and the importance of recognising relationality and people’s social infrastructures in its implementation. We use the term relationality in this paper to refer to the relationships people are embedded in that shape their wellbeing and care practices such as families, and friendships, wider communities and healthcare workers [58].

## Methods

### Study design and context

The data for this study were collected via semi-structured interviews, in the formative qualitative stage of the National Institute for Health and Care Research Alcohol use disorder and DEpression Prevention and Treatment Study (NIHR ADEPT) [63]. This was a four-year project (2020–2024) that aimed to co-design a digital resource to support the care of people with co-occurring AUD and depression in the North-East and North Cumbria region of England, UK (from here referred to as NENC). A qualitative approach was chosen as it enabled us to explore the topic in-depth from the perspectives of those involved in the delivery of care, and those receiving care, and to generate insights about issues that may not always be obvious [64]. It was our hope that these insights would help to generate new possibilities and ideas for ways digital healthcare should be developed [65]. While we included service users in the formative work conducted as part of this study, this paper uses the data gathered solely from the practitioners. Findings from patients / service users accounts has been reported elsewhere [10].

THE NENC has a population of approximately three million [66] and comprises urban, rural and coastal communities. NENC experiences higher than average levels of deprivation than other areas of England [67, 68] and, more digital exclusion than other regions. In the general population approximately 8% of households are offline compared to 5% nationally, and 32% have low levels of digital engagement compared to 28% nationally [69]. People in NENC also experience significant health inequalities compared to other regions [70]. The region has the third highest rate of common-mental illness [71] and the highest rates of suicide [72] and alcohol related morbidity and mortality in England [73]. The high levels of deprivation in NENC, and the need in relation to co-occurring AUD and depression, make it a rich case study setting for exploring the use and implementation of digital healthcare. The timing of the study, May 2021 to June 2022, followed the period of rapid change in the UK and in other developed countries in the use of telemedicine - during and following the Covid-19 pandemic.

### Sampling and recruitment

Our sampling frame comprised practitioners in NENC who deliver or commission services for adults with co-occurring AUD and depression. This included statutory (government-funded) and non-statutory care providers (charities, community and voluntary sector organisations), and local government officials in commissioning roles in local authorities and the National Health Service (NHS). Our inclusion criteria were:

- Involved in delivering or commissioning services for patients with AUD and/or depression.
- Based or working within NENC.
- Aged 18 + years.
- Willingness to participate in the project.

We adopted a purposive sampling approach [74], as we aimed to include people from across the region who had a particular interest or knowledge about care for co-occurring AUD and depression, and people with a variety of experiences of delivering or commissioning digital healthcare. We initially identified potential participants via existing contacts. As the study progressed, we incorporated a snowballing approach [74], whereby we asked interviewees to identify anyone else they knew who would meet the inclusion criteria. For all potential participants, we made an initial contact via email or telephone and included the study written information sheets. For those who expressed an interest in taking part, we contacted them, explained the study and provided an opportunity to ask questions. We re-emphasised the voluntary nature of the project. For those who were interested and provided their informed written consent, we then arranged a time for the interview.

### Data collection

Twenty-six semi-structured interviews [75] were carried out by one researcher between May 2021 and April 2022 (KJ) either using an online video conferencing platform (*n* = 21), over the telephone (*n* = 4) or face-to-face (*n* = 1). The data were generated following a topic guide that was designed by the study team to explore the current delivery of care for people with co-occurring AUD and depression in NENC and participants views about digital health technologies for this population (see additional file 1). No framework was used in the design of the guides. Participants were aware that an aim of the study was to develop a digital resource, and their accounts and views about digital health technologies were both unprompted and prompted by the researcher depending on the participant. The interviews lasted a mean average of 58 min. They were audio-recorded, transcribed by a professional transcription company and anonymized and checked for errors by the researcher.

### Data analysis

Interview data were initially analysed using stages linked to thematic analysis [76], this took place iteratively during and after the data collection process. While the data-collection was ongoing, one researcher (KJ) familiarised themselves with the data by reading and re-reading the transcripts and making notes to capture insights, with some in relation to how people were speaking about digital healthcare. Another researcher (AOD) coded three (just over 10%) of the transcripts, and they worked together to develop a coding framework which was finalised when all the transcripts were available. The coding process was primarily inductive but some deductive coding also took place to explore specific issues we were interested in such as the perceived barriers that people with AUD and depression face when accessing healthcare. Data collection ceased when it was perceived that data sufficiency was achieved for the study [77]. The transcripts were put into NVivo Qualitative Research software (v21) to support data management, and the coding framework was applied to the data. The codes were grouped into initial thematic areas shown in additional file 2.

Many of the codes and thematic areas concerned participants’ views on support for co-occurring conditions more broadly, while a sub-set related specifically to digital healthcare. To develop the interpretation that is presented here, the researchers’ used the three thematic areas with data specifically related to participants views about digital technologies: Barriers and limitations of digital technologies; Digital and Covid-19; and Opportunities of digital technologies, and developed seven themes across the data (see additional file 2). They then employed an abductive analysis [78] and considered different theoretical frameworks and concepts that could help illuminate understanding relating to the research aim. Both researchers chose the stages of care presented by Tronto and Fisher [55] for this interpretation as four phases of care were perceived to be relevant to the study focus and to fit well alongside the data. The researchers grouped the themes into the phases of care, and associated moral principles identified by Tronto and Fisher [55, 56].

### Researcher positionality

The authors are academic researchers who have experience of public health research about alcohol and substance use, and experience of the designing and studying the implementation of digital technologies in different national and international contexts. The researcher who undertook the interviews and led the analysis would describe their view about digital healthcare as ambivalent [79]. They and the co-authors have tried to maintain a stance of neutral inquisitiveness in their interpretation of the data and when developing the manuscript, with a commitment to reducing health inequalities for people with AUD and / or depression.

## Findings

Twenty-six practitioners took part in the study. Table 1 shows the job sectors participants worked in, their gender and the geographical areas of NENC they worked across. We have used quotations from participants to support the interpretation, but we have omitted their demographic information and job sectors from the quotes to help preserve anonymity.

**Table 1:** Participant characteristics

Characteristic		*N*
Gender	Male	13
	Female	13
Job Sector	NHS Alcohol (inc. dual diagnoses within mental health teams and addictions psychiatry within alcohol services)	10
	NHS Mental Health (inc. recovery colleges and women’s services)	6
	Primary healthcare	4
	Community and voluntary sector	3
	Local government	3
Geographical area of the NENC region	Newcastle and / or Gateshead	6
	Durham and / or Darlington	4
	North Cumbria	4
	South Tyneside and / or Sunderland	3
	Teesside	3
	Northumberland and / or North Tyneside	2
	NENC region-wide	4

## Caring about: attentiveness

We identified two sub-themes related to attentiveness: exclusion/inclusion and Safety.

### Digital exclusion/ inclusion

Many participants suggested that not having access to digital technology or the internet due to financial constraints was a particular challenge for the people they worked with. They noted that people often do not appear to have access to computers, or enough money to regularly update their phone credit or replace broken technology.

*One of the things we found when we moved our services online. . was people’s access to internet*,* to a decent speed on an internet. You know*,* the ability to- Some people trying to read something on your mobile phone*,* it’s impossible*,* and not everyone has a laptop or a bigger screen. Broadband speeds and not having the internet on one week and it gets cut off the next*,* because they can’t afford to pay it. [Participant 19]*

*I think it’s reasonable to say that people with comorbid depression and alcohol dependence often find themselves at the bottom of the tree when the smartphones are handed out. . And that digital poverty is quite keenly felt amongst that population. . my experience is that*,* often*,* they don’t have fancy kit. You can phone someone up*,* but yes*,* they’ll often not have a reliable number. . Maybe it’s because folks give someone else’s number or a friend’s number or that kind of thing*,* but it often seems like everything is more insecure than having a stable mobile number*,* a reliable access to the internet and a computer – I think is the barrier that I’ve come across. [Participant 16]*

Several participants also conveyed concerns that people they worked with had low digital literacy, they observed that people often did not appear to feel confident using video-conferencing software to enable them to use telemedicine. However, as Participant 24 notes in the quote below a few practitioners described they had been able to upskill some people to use telemedicine during the pandemic:

*[Our service] used Zoom calls*,* which obviously*,* creates a barrier because not everyone knows how to use them. We would try our best to teach people. As much as you can*,* having to teach people*,* while using technology*,* if they’re not confident. But we found the more people come along to it*,* and they did start learning. [Participant 2]*

Overall, many participants’ accounts suggested that they felt that telemedicine could not constitute ‘caring about’ this population because it’s use is not attentive to the digital exclusion that people are experiencing. This suggested that many participants were concerned with particularities of the care context and perceived that digital healthcare could not provide equitable access to patients who were most in need. Some participants noted that people had been given phones or tablet computers during the pandemic to help them access online support but stated that this only provided a temporary solution that they did not appear to feel was sustainable.

On the other hand, a few participants said they had observed people access technology during the pandemic and since, that they would not have expected to before the pandemic. These practitioners’ accounts suggested that this had facilitated digital inclusion and altered their perception of the usefulness of digital healthcare for some people they work with:

*Some [patients] are lucky if they have a phone*,* never mind having sufficient signal for broadband or enough money to pay for broadband. So*,* I think that can be a barrier for people for remote access. But we have the capability to [move services online] and that has been a real*,* I guess*,* benefit from COVID in a way. Something good came out of it*,* in that some of the barriers that we were concerned about we just had to get on with it when it happened. That has worked out quite well. [Participant 8]*

A few practitioners gave specific examples of where access to telemedicine had enabled people to gain support or treatment when they might otherwise not have been able to. For example, Participant 22 described how a community drug and alcohol use service had bought a smartphone for a woman with young children who lived in a rural area, which had enabled this woman to access an online mutual aid group which supported their mental health and AUD:

*The closest AA meeting to her was still a bus ride away and it was on an evening. She was relying on a neighbour to look after the kids for the two hours that she was gone. And she was still spending a couple of quid on bus fares*,* whenever she went. So*,* she got a mobile phone given to her*,* this AA group set up a digital offer where you could actually phone into the group and still get the benefit of it. So*,* she stayed at home with her kids and was saving on bus fares. It was something like*,* I don’t know*,* £3.50 a week. . over a month*,* that was a difference*,* that money could be spent on something else. She wasn’t stood at bus stops*,* pitch black evenings*,* wondering if- You know*,* all those sorts of things. [Participant 22]*

As is observed in Participants 22’s quote here, concerns about relationality were also evident in how participants spoke about attentiveness. Some participants noted that where digital healthcare was attentive to people’s relational networks or looked beyond their AUD and depression to consider their social circumstance or contexts, i.e. paid work commitments or family commitments, it could be more likely to be considered as ‘caring about’.

Also relating to digital exclusion, several participants suggested that digital technology was excluding older people, with some suggesting digital healthcare might support the inclusion of younger people. In the quote below, Participant 23, who had a particularly positive view of the value of digital technology, first described how they supported older people to use telemedicine and described what they perceived to be the future value of digital health technology for younger people:

*[To older people] I’m just like*,* “Okay*,* and let me show you something.” So*,* I sit next to them*,* right*,* and I put something in their diary*,* and I say*,* “Click join on your phone*,*” and I’m like*,* “You can see me. I’m just not in your room.” It was like a lightbulb moment. [on the other hand] The younger ones seemed to pretty much have it in hand is what I’m noticing. There’s a trend*,* yes? The older ones*,* some of them it’s a no*,* yes? They’ll ring. So*,* they like a phone call. Love a phone call*,* but they don’t necessarily engage with text messages or apps*,* but you’ve always got to be forward-thinking*,* and generations leave us at some point*,* and we have to work with the upcoming trends. [Participant 23]*

Participant 23’s account also illustrates the relational support they had given older people to use technologies. Their account, like a few others, suggested that observing digital inclusion had changed the way thought about how digital healthcare could be used in the future. While others, like Participant 8, who had observed digital healthcare seeming to help some but not all people they worked with, appeared to feel that ultimately if these technologies were excluding most people, they were not offering everything they would expect of ‘good care’.

### Safety

Many participants expressed concern that digital healthcare could not be fully attentive to their concerns about the safety, in particular the high risk of suicide in patients with AUD and depression. To illustrate, the following quote illustrates Participant 14’s apparent apprehension about whether telemedicine would enable them to observe any subtle changes in patients who might indicate they were at risk of suicide:

*If we say*,* “Okay*,* there’s this brilliant thing [digital app]. You can start using that and I’ll see you in a couple of weeks’ time” Even the person themselves might not notice early warning signs. Either the depression is getting worse*,* or the drinking is increasing. Even just the level or frequency or the alcohol is getting stronger*,* that’s where… That’s our only insight*,* is having a visible face-to-face meeting with somebody noticing signs and symptoms. It’s just taking away that human element of gut instinct or observations really. [Participant 14]*

Another participant who worked in a charity with people with experience of trauma, strongly conveyed that they did not think telemedicine was appropriate for the population they worked with and would not consider using telemedicine because of concerns about risk:

*You know*,* if we think about the nature of when people drink*,* it’s normally because they’re in a state of distress and there is a lot of emotion there anyway*,* so it becomes very difficult to even monitor that or to boundary that. So yes*,* that is quite difficult. So*,* I think- I’m not aware that we supported anyone who was drinking heavily during COVID on an online basis. [Participant 19]*

While it was most common that participants expressed concerns about digital technology being able to accommodate risks or safety, a small number of participants discussed situations where m-health could support managing the safety of patients. For example, a few participants discussed the usefulness of safety planning apps for mobile phones. A common challenge reported by people with depression and AUD and the practitioners who work with them is that mental health crisis services often will not support people in mental health crisis who are intoxicated. One participant discussed the potential value of using telemedicine to provide remote mental health crisis support for people who are intoxicated:

*Because if a [practitioner] was worried the person drinking might be aggressive or not be able to be in the building because of it*,* which I know is a thing that I’ve heard. . Yes*,* so I think that would be a great thing. To still be able to get an appointment. So*,* if they’re used to being told*,* “If you’ve had a drink*,* you can’t come to your appointment*,*” they would be able to still have that appointment. [Participant 21]*

Thus, while it was more common that participants expressed concerns about safety and digital healthcare, a minority suggested it could potentially help to improve safety – meaning that digital healthcare had the capacity to be both more inclusive and attentive to people’s needs in a way that in-person care might not.

## Caring for: responsibility

One theme related to responsibility was evident across almost all participants accounts; most practitioners stated that digital healthcare should never be the only option available to people. Concerns about this seemed to stem from participants’ awareness that people with AUD and depression need in-person support and to feel socially connected.

### Digital healthcare should always be part of a wider infrastructure of support

Almost all the participants reported that digital technologies should never be seen as a replacement for in-person care. Rather it should be considered as in addition to a wider infrastructure of in-person care. Participant 13 who conveyed that they did not believe the population liked digital healthcare, made a typical comment that they perceived digital technology should never be offered without the option of in-person support:

. *. .we’re often*,* or sometimes*,* talking about people for whom technology is loathed and actually you can’t just. . That can’t be the only thing on the menu. [Participant 13]*

A few practitioners overtly expressed the concern that in a context where policy makers were trying to reduce costs, mobile phone apps and websites could be seen as passing the responsibility to someone else and not offering the type of care that was needed.

*[Digital healthcare] can be a bit of a sort of substitute for actually genuine attempts to engage [patients]. So*,* you would have to make sure that it was being done with standard care*,* or somehow complementing existing care*,* rather than being*,* “There’s some great news*,* guys. We’ve got this new resource*,* which means we don’t have to see all of our depressed people with alcohol problems anymore*,* because there’s a website for that*,*“. . [Participant 5]*

Although Participant 5 uses humour here, their comment indicates an underlying concern that where technology was used as a means of transferring responsibility to patients to manage their own condition, this could represent a lack of care.

Many participants’ accounts also suggested that they perceived that for digital healthcare to be used and realised in this population people needed help from practitioners or family / or friends and that a healthcare worker should take responsibility for supporting people to access it. Participant 23’s example above of supporting people to use telemedicine was an example of this. This point was also conveyed by others, for example Participant 1 said:

*I think*,* not just leaving people to access digital technology as well. It’s about having opportunities to check in with individuals. [Participant 1]*

Thus, both websites, mobile phone apps and telemedicine alone were not seen as taking responsibility for patients if they were not appropriately supported to use them.

## Care giving: competence

Two areas in relation to competent digital care for the population were highlighted: the need for embodied interactions; and the need to be flexible and adaptable.

### Embodiment and physicality

Following on from the concern that digital healthcare should not be the only option for people, many participants accounts illuminated their perception that competent care giving for people with co-occurring AUD and depression needed to include some physical or embodied contact, which it was noted digital healthcare – whether telemedicine or apps/websites - could not provide.

*[Digital healthcare is] not the same. That’s how I feel from a personal point of view. You’re missing that touch of empathy really shining through*,* and congruence*,* and all those basic*,* not counselling*,* because that’s not what I’m trying to say*,* but let’s be honest in our role we’ve all got basic counselling skills. you can’t put those across when it’s through video calls*,* or even phone calls. It’s a lot harder to do that. [Participant 26]*

Many patients were perceived to have limited access to familial and relational networks and practitioners’ discussions of the need for these embodied aspects stemmed from a wider concern that many people in the population were often socially isolated and had experienced trauma:

*We have to be careful*,* because there are going to be issues around social isolation. But there would never just be a digital offer*,* it should always be a combined offer. [Participant 22]*

*I sat in the [group] and realised the importance of physicality when you’re dealing with people with deep trauma. [Participant 7]*

Several participants spoke about the physical and embodied aspects of their work and how this could be used to convey empathy and compassion for the challenging circumstances that many people were experiencing. For example, Participant 24 explained:

*I found that in the fellowship meetings when they all went to Zoom*,* a lot of people stopped going. A lot of people have said*,* “I’m missing me meetings*,* just catching up with somebody.” A lot of the meetings are very physical*,* like before and after the meetings in the 12-step programme they give each other a hug. . [Participant 24]*

Thus, here we see even where people did have access to technology without the physical component it was not seen to be offering the care that people needed. Thus, improving access to technology alone would not offer appropriate care.

### Flexible and adaptable

Several participants emphasised that people with AUD and depression often lead complex lives which a structured and rigid approach cannot accommodate. There were concerns from some participants that digital healthcare – in particular websites and apps – were not responsive to people, this also related to concerns about safety. This is illustrated in Participants 2’s point:

*The other thing of course with alcohol is that it is potentially fatal. . I am able to do some basic kind of tapering advice. . I think you would be very*,* very hard pressed*,* or you would have to do it very clinically*,* very carefully*,* to do an alcohol app that takes into account risks of reduction. . that would be tricky. [Participant 2]*

Conversely, other participants suggested that a perceived value of digital healthcare was that it could be flexible. Several practitioners gave an example where they had observed digital healthcare as supporting people at times that they would not otherwise have been supported and needed care most:

*I think where people’s*,* maybe*,* lives may be a little bit more chaotic as a result of heavy alcohol use and having structured set times for when you access [in-person] appointments might not work for an individual. I think having some flexibility*,* and I think that’s where digital options would benefit that individual*,* that they can access support or access materials at a time that’s convenient for them*,* when they’re in the right*,* sort of*,* place to do so. So*,* I think that flexibility is definitely a positive. [Participant 1]*

Other participants emphasized how digital healthcare could potentially fit more easily alongside patient’s family and work commitments. To illustrate, Participant 13 who was also involved in the mutual help community described how a friend used telemedicine when they were at work:

*A friend of mine works on a building site and he drives a huge*,* big digger thing. He often has his phone next to [him]. . .So*,* he’ll have company whilst he’s at work. He’s not attending an appointment. This is just part of his life as he’s going about his daily job. [Participant 13]*

Thus, again we see the nuance in people’s accounts with people mixed in the extent to which they seemed to perceive that digital healthcare could provide competent care to respond to people’s needs.

## Care receiving: responsiveness

Concerns about responsiveness related to honesty or disclosure when interacting online, and motivation.

### Honesty / disclosure

Some participants expressed concern that patients might try to hide things when using telemedicine that they or another practitioner would be able to notice face-to-face:

*Sometimes I guess there are concerns that people could be intoxicated*,* and they may communicate about what is happening to them in an area where they shouldn’t do*,* and you are trying to assess that. Or obviously they underplay some of their features of intoxication that would be apparent in a face-to-face. [Participant 8]*

*You’re anonymous*,* and you can choose not to contribute. . .You could choose to take a backseat and switch off*,* literally. I think there’s something to be said for the live experience that it draws different things out. [Participant 13]*

However, a small number of participants suggested that patients might be more likely to engage online because of the stigma that surrounds addiction because it could help retain their anonymity. Or as Participant 22 noted, interacting remotely might be less intimidating than working in a group face-to-face:

*We just expect people to come here. “Come into this group and tell incredibly personal things about yourself to these strangers.”. . So*,* [online support] gets around that a little bit*,* I think*,* people can feel maybe a little bit safer. [Participant 22]*

### Motivation

Several participants highlighted the impact that having AUD and depression could have on people’s levels of motivation and there were concerns that people might not be motivated enough to engage with online tools or might stop engaging after a period. Participant 14 expressed a concern about how individual motivation and digital exclusion due to their material circumstances could impact on how patients would engage with digital healthcare:

*I think*,* for somebody who is drinking really heavily or it’s going up and down and experiencing… Like having an impact with mood disorder… It’s really hard*,* you need to be quite self-motivated and tech-savvy and… you’re drinking and you’re in a situation where there’s disadvantage and you haven’t got a lot of money*,* if your money is going on alcohol chances are you don’t have an internet connection or a laptop so opportunity is probably not there. Then*,* in terms of capability… They might have capability when they’re sober but when you’ve had a good drink*,* chances are that capability is dropping. Keeping up with that work*,* accessing the thing that’s available… [Participant 14]*.

Thus, this example illustrates the connection between the different stages of care and the need to recognise that these stages overlap in how people think about the usefulness of digital healthcare for this population.

## Discussion and conclusion

This study provides insight into areas where digital healthcare is considered ‘good care’, and where it can be considered to fall short, by drawing on the perspectives and experiences of the multi-disciplinary workforce who support people with co-occurring AUD and depression. By using Tronto and Fisher’s conceptualisation of the care process [55, 56] and the broad principles of ethics of care theory [57], we extend existing work in this space by illuminating that different dimensions of care are needed for practitioners to feel like digital healthcare is caring for this often-underserved population with integrity. The similarities we have identified in the concerns perceived by a varied group of practitioners point to areas that those involved in developing and implementing health technologies for people with co-occurring AUD and depression need to consider if any digital interventions are to be not only implementable, but morally acceptable to practitioners who provide care to this population.

The findings indicated that the workforce perceived that digital technologies could have a role in care provision for some patients, giving examples of digital inclusion. Often based on their own observations and experiences of working with patients since the pandemic, several practitioners suggested that digital healthcare had the potential to be more flexible and person-centred than the rigid way in-person care for this population is currently configured in the UK. Some practitioners discussed times where digital healthcare had enabled patients to access services who would not otherwise have been able to. A few practitioners noted that digital healthcare can potentially lessen stigma for patients with co-occurring AUD and depression, as it can support anonymity, and thus make it more likely for them to engage in treatment. These positives of digital care have been highlighted by other empirical studies in mental health and / or alcohol use contexts [32]. They also echo policy rhetoric about the value of digital healthcare more generally [35]. However, by viewing the themes through a lens which illustrates the interconnectedness of the dimensions of care, our study extends the literature to highlight some key factors that need to be considered if digital care is to be considered good care by practitioners, whose views and experiences are likely to contribute to their own and patients’ future engagement with digital healthcare.

### Implications for the development of digital healthcare for people with co-occurring AUD and depression

Unsurprisingly, a key concern raised by many practitioners related to digital exclusion. Most had experienced their patients being unable to access digital healthcare because they lacked access to the equipment or the internet due to their adverse material circumstances. Low digital literacy was also observed to be common in the population. Thus, our study further indicates that if we continue to develop digital technologies for this population without considering the economic contexts of patients, the use of digital health technologies has the potential to create further inequalities [42]. Researchers and service developers in other settings have tried to overcome material inequalities and low levels of digital literacy when developing interventions [45, 61]. In the UK there are now various toolkits and guidelines in place to support digital inclusion [80, 81]. We echo these in noting the need to always consider people’s long-term access to equipment such as phones, tablet computers and personal computers, and people’s long-term access to the internet. Moreover, it is also crucial that individual levels of digital literacy should always be considered in the design and implementation of digital healthcare interventions. Our study is supportive of scholarship which advocates for involving the end user (both patients and practitioners), and especially the most marginalised groups, in the development of digital health technologies [65].

An overarching finding from this study related to the importance of recognising people’s relational contexts when implementing digital technologies, which links back to the wider tenets of ethics of care theory. Practitioners’ accounts revealed that they had observed patients needing in-person support from healthcare professionals to initiate and maintain their use of digital technologies. Moreover, due to levels of social isolation common in this population, there was concern that removing access to human infrastructure of care could be detrimental. As such, most practitioners suggested that digital healthcare should only be used as part of a hybrid model of care, where support could be adapted to suit the specific needs of the individual patient, including where appropriate, face-to-face interaction [34], and would therefore be reluctant to see unguided support [32]. Researchers in other areas of healthcare have found that m-health works best for those who already have support from healthcare professionals and family members, as opposed to those who are socially isolated [60, 82]. It is paramount that those developing digital health technologies start with relationality and inequality at the centre ; this ethos aligns closely with an ethic of care where attention to inequalities and relationality is fundamental [58]. A key implication of the work is that for people with AUD and depression who are often lacking in relational support [10], social isolation, should be recognised as a key challenge to the roll out of digital healthcare in this population. Future studies of the development or implementation of digital interventions for people with AUD and depression need to consider their existing relational infrastructures as a matter of priority.

Concerns about not being able to manage the safety of patients with AUD and depression when using digital technologies were highlighted as a further area where digital technology might not be perceived as attentive to the specific needs of the population. Other studies have suggested ways that concerns about safety could be mitigated. For example, by ensuring people are assessed for their suitability for using digital technologies [34] or by including alerts on apps and websites [83]. Provider-guided technologies have also been perceived to be valuable by practitioners in other mental health contexts [32], with other literature highlighting the importance of ensuring there is always in-person support available alongside any digital technologies [80]. Therefore, a final key implication is that developing and implementing digital technologies for people with AUD and depression need to ensure that there are effective risk planning procedures in place.

### Strengths and limitations

This exploratory qualitative study was undertaken in one geographical region of England; the findings might not be representative of practitioners in other regions of England or other countries. However, the similarities of the sub-themes with other national and international studies of different types of healthcare practitioners give us confidence that the findings could be transferable. Moreover, the use of the theoretical framework which has been used in other contexts (e.g. [84, 85]) adds depth to the analysis and strengthens our confidence in the applicability and relevance of the interpretation beyond the study setting. Additionally, while we sought to include the perspective of practitioners from a range of services, some types of health and social care practitioners who work with people with AUD and depression have not been included in the study, for example occupational therapists and social workers. Unpaid carers were also not included in the study who provide an important role in providing care for this population. Critically, future design and implementation work should consider the views and experiences of patients with AUD and depression themselves.

## Supplementary Information

Below is the link to the electronic supplementary material.

Supplementary Material 1

Supplementary Material 2

## Data Availability

The datasets generated and/or analysed during the current study are not publicly available due to the nature of the location and field of healthcare making participants potentially identifiable via full interview transcripts. However, data in analysed form are available from the corresponding author on reasonable request.
