# Supplementary material for: When do health and social care practitioners consider digital healthcare to be ‘good care’ for people with co-occurring alcohol-use disorder and depression? a qualitative analysis of practitioners’ accounts
Source: BMC Health Serv Res. 2025 Dec 23;26:123. doi: 10.1186/s12913-025-13883-3 (PMC12836889; doi:10.1186/s12913-025-13883-3)
Supplement: Supplementary file 1 — Supplementary Material 1 [file 12913_2025_13883_MOESM1_ESM.docx]

1. A table to show the broad thematic areas and codes generated from the full data

| **BROAD THEMATIC AREA** | **CODE** |
| --- | --- |
| **Assessing need and meeting eligibity criteria** |  |
|  | Alcohol as barrier to mental health care |
|  | Assessment as barrier |
|  | Crisis assessment criteria |
|  | Focus on severe cases |
|  | Uncertainty in discharge planning |
| **Challenges to delivering good care** |  |
|  | Family and peer networks |
|  | Fixed address or housing |
|  | Group based work |
|  | High risk patient group |
|  | Impact of social norms around alcohol |
|  | Impact of stigma |
|  | Information systems and data |
|  | Lack of awareness of services |
|  | Lack of mutual aid culture |
|  | Large geographical area |
|  | Low self-worth in population |
|  | Managing care in chaotic patient group |
|  | Managing expectations or no quick fix |
|  | Motivation to change |
| **Relationship between alcohol and mental health** |  |
|  | Common approach |
|  | No separation between drinking and mental health |
|  | Role for dual diagnosis |
| **Stigma and social norms** |  |
| **What good care looks like** |  |
|  | Awareness of relevant services |
|  | Compassionate care |
|  | Continuity in provider |
|  | Drawing on lived experience |
|  | High support high challenge |
|  | Inclusive care |
|  | Joined up care |
|  | Medical and social care skills |
|  | Persistent |
|  | Personalised care |
|  | Promoting self-advocacy |
|  | Third sector for complex service users |
|  | Trust and relationships |
|  | Value of peer support |
| **Capacity and responsiveness of services** |  |
|  | Access to support when its needed |
|  | Impact of COVID on care |
|  | Inadequate resources |
|  | Lack of OR focus on prevention |
|  | Negative attitudes to peer support |
|  | Nowhere to put patients |
|  | Reduced availability of care |
|  | Waiting lists |
| **Fragmented and inconsistent services** |  |
|  | Changes in government level strategy |
|  | Changes in provider |
|  | Disjointed care and work practices |
|  | Geographic boundaries as barrier |
|  | High staff turnover |
|  | Need for standardised care |
|  | Passed between services |
|  | Regional disparity in provision |
|  | Service decommissioned |
| **Integration and relationship building** |  |
|  | Accountability and guidelines |
|  | Communication and feedback |
|  | Information sharing |
|  | Involving people with lived experience |
|  | Joined-up work practices |
|  | Knowledge of local populations |
|  | Need for whole system approach |
|  | Relationships across services |
|  | Shared goals |
| **Managing Risk** |  |
| **Skills and expertise** |  |
|  | Evidence based interventions |
|  | System knowledge |
|  | Role of digital technology |
| **Barriers and limitations of digital technologies** |  |
|  | Concerns about confidentiality |
|  | Financial and access |
|  | Lack of capacity or motivation to engage (and expectations of) |
|  | Managing risk |
|  | Practitioners skills and confidence |
|  | Relationship building |
|  | Technical issues |
|  | Value of face-to-face |
| **Digital and Covid-19** |  |
| **Opportunities of digital technologies** |  |
|  | Accessible and convenient |
|  | At own pace |
|  | Confidentiality and anonymity |
|  | Hybrid care model |
|  | Information system - recording |
|  | Link to peer support |
|  | Promotes self care |
|  | Reduce pressures on practitioners |
|  | Supports safety and risk |
|  | Waiting list support |

1. A table to show the key themes generated from the data related to digital technologies

| **THEME** | **CODE** |
| --- | --- |
| **Digital Exclusion / Inclusion** |  |
|  | Financial and access |
|  | Technical Issues |
|  | Accessible and convenient |
| **Safety** |  |
|  | Managing risk |
|  | Supports safety and risk |
| **Digital as part of a wider infrastructure of support** |  |
|  | Hybrid care model |
| **Flexible and adaptable** |  |
|  | Accessible and convenient |
|  | At own pace |
| **Embodiment and physicality** |  |
|  | Value of face-to-face |
|  | Relationship building |
| **Honesty / disclosure** |  |
|  | Confidentiality and anonymity |
|  | Managing risk |
| **Motivation** |  |
|  | Lack of capacity or motivation to engage (and expectations of) |
